# Association of polygenic risk score with response to deep brain stimulation in Parkinson’s disease

**DOI:** 10.1186/s12883-023-03188-5

**Published:** 2023-04-04

**Authors:** Esther Yoon, Sarah Ahmed, Ryan Li, Sara Bandres-Ciga, Cornelis Blauwendraat, Irene Dustin, Sonja Scholz, Mark Hallett, Debra Ehrlich

**Affiliations:** 1 Parkinson’s Disease Clinic, Office of the Clinical Director, National Institute of Neurological Disorders and Stroke, NIH, 7D37 10 Center Dr, Bethesda, MD USA; 2 Neurodegenerative Diseases Research Unit, National Institute of Neurological Disorders and Stroke, NIH, Bethesda, MD USA; 3 Molecular Genetics Section, Laboratory of Neurogenetics, National Institute of Aging, NIH, Bethesda, MD USA; 4 Department of Neurology, Johns Hopkins Medical Center, Baltimore, MD USA; 5 Human Motor Control Section, National Institute of Neurological Disorders and Stroke, NIH, Bethesda, MD USA

**Keywords:** Deep brain stimulation, Parkinson’s disease, Polygenic risk score

## Abstract

**Background:**

Deep brain stimulation (DBS) is a well-established treatment option for select patients with Parkinson’s Disease (PD). However, response to DBS varies, therefore, the ability to predict who will have better outcomes can aid patient selection. Some PD-related monogenic mutations have been reported among factors that influence response to DBS. However, monogenic disease accounts for only a minority of patients with PD. The polygenic risk score (PRS) is an indication of cumulative genetic risk for disease. The PRS in PD has also been correlated with age of onset and symptom progression, but it is unknown whether correlations exist between PRS and DBS response. Here, we performed a pilot study to look for any such correlation.

**Methods:**

We performed a retrospective analysis of 33 PD patients from the NIH PD Clinic and 13 patients from the Parkinson’s Progression Markers Initiative database who had genetic testing and underwent bilateral subthalamic nucleus DBS surgery and clinical follow-up. A PD-specific PRS was calculated for all 46 patients based on the 90 susceptibility variants identified in the latest PD genome-wide association study. We tested associations between PRS and pre- and post-surgery motor and cognitive measures using multiple regression analysis for up to two years after surgery.

**Results:**

Changes in scores on the Beck Depression Inventory (BDI) were not correlated with PRS when derived from all susceptibility variants, however, when removing pathogenic and high-risk carriers from the calculation, higher PRS was significantly associated with greater reduction in BDI score at 3 months and with similar trend 24 months after DBS. PRS was not a significant predictor of Unified Parkinson’s Disease Rating Scale, Dementia Rating Scale, or phenomic and semantic fluency outcomes at 3- and 24-months after DBS surgery.

**Conclusions:**

This exploratory study suggests that PRS may predict degree of improvement in depressive symptoms after DBS, though was not predictive of motor and other cognitive outcomes after DBS. Additionally, PRS may be most relevant in predicting DBS outcomes in patients lacking pathogenic or high-risk PD variants. However, this was a small preliminary study and response to DBS treatment is multifactorial, therefore, more standardized high-powered studies are needed.

**Supplementary Information:**

The online version contains supplementary material available at 10.1186/s12883-023-03188-5.

## Background

Deep brain stimulation (DBS) is a well-established surgical treatment option for select patients with Parkinson’s disease (PD) and can effectively improve motor complications, dyskinesia, and quality of life. Multiple factors can influence DBS outcomes, including age, disease duration, co-morbidities, levodopa responsiveness, symptom severity, cognitive and psychiatric impairment, electrode placement, and post-operative programming [1, 2]. Recent studies have suggested that genetic factors may also impact response to DBS. Glucocerebrosidase (*GBA*) mutation carriers have worse cognitive performance than non-carriers after DBS surgery [3, 4], and parkin (*PRKN*) carriers have greater levodopa-equivalent daily dose (LEDD) reduction after DBS when compared to non-carriers [5, 6]. Additionally, patients with PD due to the common p.G2019S mutation in Leucine-rich repeat kinase (*LRRK2*) have excellent motor benefit from DBS, while *LRRK2* p.R1441G mutation carriers exhibit worse outcomes than non-carriers [7].

Genome-wide association studies (GWAS) have identified common genetic variants associated with risk of disease. A recent PD meta-GWAS analyzing 37,688 cases, 18,618 UK Biobank proxy-cases, and 1.4 million controls identified 90 risk variants, explaining 16–36% of heritable risk for PD in European populations [8]. A PD-specific polygenic risk score (PRS), which is a weighted score based on a person’s allelic status of all variants found through GWAS and the magnitude of effect for each variant using reference summary statistics, has been associated with disease risk. More recently, PD-PRS was also found to correlate with PD age of onset and progression of motor and cognitive symptoms [9]. Although prior studies examined associations between monogenic forms of PD with DBS outcome, monogenic disease accounts for less than 5% of the PD patient population. Here, we explored the relationship between cumulative genetic risk burden (PRS) and DBS outcome in PD patients.

## Methods

### Patient selection & assessment

We performed a retrospective analysis of 33 unrelated European-ancestry PD patients who underwent bilateral subthalamic nucleus (STN) DBS surgery (Activa PC Deep Brain Stimulation system, 3389 electrodes; Medtronic) and completed at least two years of follow-up at the U.S. National Institutes of Health (NIH). Prior to surgery, all participants underwent screening, which included medical history, physical examination, blood test, Unified Parkinson Disease Rating Scale (UPDRS), MRI scan, and neuropsychological testing. The neuropsychological evaluation administered by a neuropsychologist included the Mattis Dementia Rating Scale-2 (DRS), Beck Depression Inventory-2 (BDI), and verbal fluency tests.

The cases were selected for surgery based on the requirement of at least 18 years of age, ability to undergo MRI, and ability to provide informed consent. Patients were included if they had a diagnosis of PD based on UK Brain Bank Criteria [10] and confirmed by a movement disorders neurologist in the NIH Parkinson’s Disease Clinic, with at least 30% improvement on UPDRS III with levodopa, and the presence of intractable motor fluctuations, insufficient duration of action with dopaminergic medications, or unacceptable medication side-effects. Patients were excluded if they had a clinically significant medical condition which could increase the risk of pre- or post-operative complications, such as uncontrolled hypertension (blood pressure > 170/100) or an unstable cardiac or respiratory condition. Patients were also excluded if they had active depression, identified by self-report or Beck Depression Inventory-2 score ≥ 20, or dementia, as evidenced by formal neuropsychological evaluation, or Mattis Dementia Rating Scale-2 score < 128. Secondary or atypical parkinsonism cases were also excluded. Follow-up examinations were completed by the Parkinson’s Clinic team from 1- to 24-months post-surgery, and neuropsychological testing and MRI scans were performed during the 3- and 24-month visits. Post-operative UPDRS scoring was conducted with patients both on-medication and on-stimulation. This study was approved by the NIH Combined Neuroscience Institutional Review Board, and written informed consents were obtained from all patients.

In addition, DBS, genetic, and Movement Disorder Society (MDS)-UPDRS data from an additional 13 European-ancestry PD patients from the Parkinson’s Progression Markers Initiative (PPMI) were included in the analysis. Further details regarding PPMI can be accessed on the PPMI website (www.ppmi-info.org).

### Whole genome sequencing and quality-control

Briefly, whole-blood DNA samples were prepared using the Illumina’s TruSeq PCR Free library preparation workflow and sequenced using the HiSeq X Ten Sequencer, per the manufacturer’s protocols, and processed following the Centers for Common Disease Genomics pipeline [11]. Details on the whole genome sequencing (WGS) and quality control methods are available on the PPMI website (https://ida.loni.usc.edu/pages/access/geneticData.jsp).

Using PLINK v.1.9 [12], quality control steps excluded samples with call rates less than 95%, heterozygous outliers with an F cut-off between -0.15 and 0.15, sex mismatches between the reported and genotypic sex, and genetically related individuals whose pair-wise kinship coefficients exceeded 0.125 (indicating second-degree relatives or closer). Duplicate and related samples were checked using the King v2.1.3 kinship tool [13]. Following the Broad’s implementation of the functionally equivalent standard pipeline for sequence and alignment related quality control, each sample’s mean sequence depth (< 30X), contamination rate (> 2%), and single nucleotide variant count (< 3 SD) based on the sample’s genomic vcf were inspected. To correct for population stratification, all samples of non-European ancestry were excluded in future analyses after a principal component analysis (PCA) on the first two PCA scores, comparing PD samples to participants of the International HapMap3 Project [14].

### Genetic analysis

PRS were calculated in PLINK v1.9 using 90 variants that displayed independent signals and genome-wide significance in the latest PD meta-GWAS [8]. Risk allele dosages were counted (denoted as 2 for homozygous for the alternate allele, 1 heterozygous and 0 homozygous for the reference allele) and a PRS was generated across all variants. All variants were weighted by their published odds ratios, giving greater weight to alleles with higher risk estimates [15]. PRS were then transformed into Z-scores. Pathogenicity of variants were established using the classification of disease-causing missense (DM) mutations in Human Gene Mutation Database (HGMD) and a clinical significance of pathogenic in ClinVar [16, 17].

### UPDRS score conversion & LEDD calculation

To combine the NIH and PPMI cohorts, the UPDRS III scores from the NIH samples were converted to the MDS-UPDRS III scores using a previously published linear conversion [18]. LEDD was also calculated using a standard guideline [19].

### Statistical analysis

All statistical analyses and visualizations were performed in R version 3.6.1 [20]. After quality control and excluding non-STN-DBS patients, a total of 46 (33 NIH, 13 PPMI) subjects remained for analysis. Independent t-tests and Fisher’s exact tests were used to compare clinical characteristics of the NIH and PPMI cohorts. To investigate the association between PRS and DBS outcome, a multiple regression model was used with PRS and MDS-UPDRS III and UPDRS I, II, and IV, LEDD, and cognitive test scores, while adjusting for age at surgery, disease duration, sex, and baseline clinical score. Outliers with baseline values greater than 1.5 times the interquartile range were removed from the analysis. Given the exploratory nature of the study, a *p*-value < 0.05 was considered significant. Associations are expressed with beta coefficients, representing a change in the DBS outcome measure with one unit change in the independent variable.

Since some pathogenic variants are known to individually confer a high risk of developing PD, possession of any of these variants can substantially increase the polygenic risk score in an individual. However, the polygenic risk score may not be as relevant in these cases since the disease risk is likely most related to the single pathogenic variant rather than polygenic risk. Therefore, two additional analyses were conducted to assess whether our results were driven by pathogenic variants (*GBA, LRRK2*, and *PRKN* mutation carriers). For the first analysis, high-risk variants in the *GBA* (rs76763715 p.N370S, rs35749011, rs114138760), and *LRRK2* (rs34637584 p.G2019S) were removed from the PRS calculation. For the second, all pathogenic or high-risk variant carriers were excluded in our model.

Complete results from the secondary analysis are reported in Supplementary File 1. Pre-operative genetic and clinical characteristics of the study cohort are available in Table S1, Figs. S1 & S2. Analyses of associations between PRS and pre-surgical characteristics can be found in Table S2.

## Results

PRS was not a significant predictor of Unified Parkinson’s Disease Rating Scale (UPDRS) I, II, and IV, Levodopa-equivalent daily dose (LEDD), Dementia Rating Scale (DRS), phonemic fluency, or semantic fluency outcomes at 3- and 24-months post-surgery (Table S3). Beck Depression Inventory-2 (BDI) outcome was not associated with PRS derived from all 90 GWAS variants. However, when *GBA* and *LRRK2* variants were excluded from the genetic risk calculation, we found PRS trending with change in BDI score at 3-months (*P* < 0.099, beta = -1.36, SE = 0.79) and 24-months post-surgery (*P* = 0.057, beta = -2.34, SE = 1.16), with higher genetic risk carriers exhibiting greater reduction in post-surgical BDI score.

**Fig. 1:**
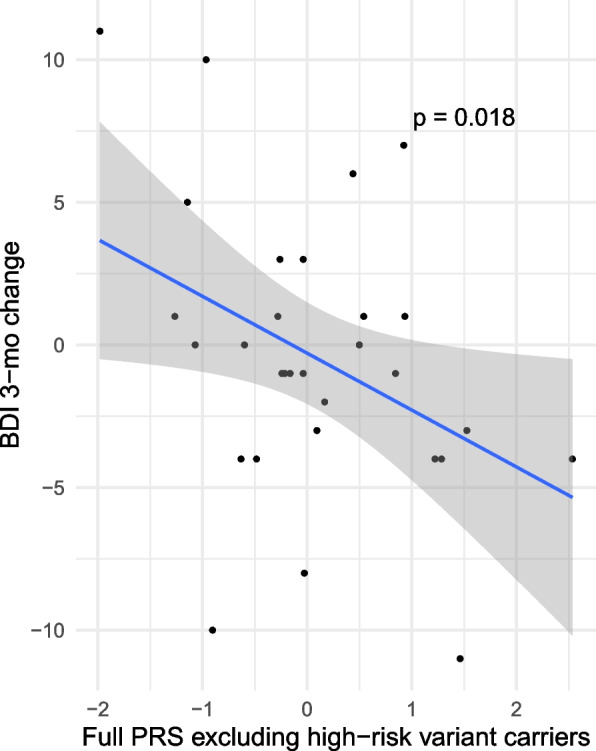
PRS excluding high-risk variant carriers and change in BDI 3-months post-surgery

Additionally, analysis excluding all pathogenic and high-risk variant carriers found higher PRS to be significantly associated with greater reduction in BDI score at 3-months (*P* = 0.018, beta = -2.16, SE = 0.85) (Fig. 1) and trending in the same direction for BDI outcome 24-months after DBS (*P* = 0.074, beta = -1.90, SE = 1.01).

## Discussion

While previous studies have reported that PRS can be useful in predicting age of onset and disease progression in PD, our findings suggest that it may not be the strongest predictor of DBS outcomes. In our study, while no associations were found between PRS and motor and most cognitive outcomes after DBS, we found PRS to be associated with change in depressive symptoms when controlling for pathogenic and high-risk variants and carriers. When high-risk *GBA* and *LRRK2* variants were removed from the PRS calculation, and when all pathogenic or high-risk variant carriers were excluded from our model, PD patients with a higher genetic risk burden showed greater improvement in depressive symptoms after DBS than those with a lower PRS. However, when pathogenic variants were included in the PRS calculation and pathogenic variant carriers were included in the model, the association between PRS and change in depression (BDI) score was not present. It is possible that the correlation between PRS and change in BDI scores after DBS was only significant when excluding pathogenic and high-risk variants because, the strength of risk associated with these variants may cause PRS to be heavily attributed to a single variant rather than cumulative risk, and make the use of a calculated risk score not relevant as a predictive score in these situations. Additionally, it is also possible that carriers of high-risk or pathogenic variants associated with depression (eg. *GBA* (3,4)) are less likely to improve cognitively after DBS. Alternatively, certain variants may be associated with less cognitive impairment, and these carriers may have lower levels of depression prior to DBS and require a higher degree of change after surgery to achieve significance.

While exploratory, our study suggests that PRS may not be as relevant for carriers of PD-related pathogenic variants such as GBA p.N370S or LRRK2 p.G2019S and other PD high risk variants on predicting DBS treatment response and may be confounding the PRS analysis results. It is possible that the predictive effects on DBS outcomes for carriers of PD-related pathogenic variants and high-risk variants is best predicted by the genotype, as suggested in prior studies, while PRS might be more relevant in predicting DBS outcomes in cases of idiopathic PD lacking pathogenic or high-risk variants. For future studies on the role of PRS on DBS-treatment response, models should control for PD-related pathogenic variants and high-risk variant carriers.

There were several limitations to our study. Our study size was small due to limited availability of patient data with bilateral STN-DBS, WGS, and comprehensive motor and neuropsychological follow-up for two years. While inclusion of data from PPMI increased our study size, it introduced additional confounds related to less standardization. Corrections for multiple testing was not performed given the exploratory nature of our study and small sample size. Additionally, as a retrospective analysis, post-DBS results were only available in the on-stimulation/on-medication conditions. Future prospective studies with larger sample size and inclusion of on-stimulation/off-medication conditions would enable expanded comparisons. Further, inclusion of a control group of comparable PD patients without DBS could better explore whether variability in DBS outcome is due to the interaction between genetic predisposition and stimulation or differential progression attributed to genetic risk burden.

Another important limitation was the selective exclusion of patients with cognitive impairment and non-European patients. A cutoff DRS score was used to exclude patients with baseline cognitive impairment which could increase surgical risk. However, we may not have found associations between PRS and cognitive outcome measures due to pre-selection of patients and limited variability in baseline UPDRS I and DRS scores. To improve our understanding of how genetic risk affects non-European populations, it will be important to study more diverse patient populations to determine if these findings can be generalized to all PD patients, regardless of ancestry.

## Conclusion

In conclusion, our exploratory study suggests that while genetic risk burden may predict degree of improvement in depressive symptoms after DBS, it does not predict DBS motor and other cognitive outcomes. Our results also suggest that use of the PRS in predicting DBS outcomes may be most relevant in idiopathic PD cases lacking pathogenic or high-risk PD-related variants, while in cases with pathogenic and high-risk variants, the specific genotype may be more important in predicting outcomes rather than the PRS. However, response to DBS treatment is complex and multifactorial, therefore, high-powered studies with more standardized measures are needed to better elucidate whether PRS should be considered in pre-operative screening when determining DBS candidacy in PD.

## Supplementary Information

**Additional file 1:** **Table S1.** Pre-operative genetic and clinical characteristics of the STN-DBS study cohort. **Table S2.** Associations between polygenic risk score with baseline clinical and neuropsychological testing measures. **Table S3.** Associations between polygenic risk score with 3- and 24-month post-surgical clinical and neuropsychological testing measures. **Figure S1.** Polygenic risk score by genotypic subgroups. **Figure S2.** Polygenic risk score excluding GBA and LRRK2 variants by genotypic subgroups.

## Data Availability

The data that support the findings of this study are available from PPMI repository and corresponding author/NIH upon reasonable request.

## Abbreviations

DBS: Deep brain stimulation
PD: Parkinson’s Disease
GBA: Glucocerebrosidase
PRKN: Parkin
LEDD: Levodopa-equivalent daily dose
GWAS: Genome-wide association studies
PRS: Polygenic risk score
STN: Subthalamic nucleus
NIH: National Institutes of Health
MDS-UPDRS: Movement Disorders Society—Unified Parkinson's Disease Rating Scale
PPMI: Parkinson’s Progression Markers Initiative
BDI: Beck depression inventory-2
