# Supplementary material for: Association of polygenic risk score with response to deep brain stimulation in Parkinson’s disease
Source: BMC Neurol. 2023 Apr 4;23:143. doi: 10.1186/s12883-023-03188-5 (PMC10071605; doi:10.1186/s12883-023-03188-5)
Supplement: Supplementary file 1 — Additional file 1: Table S1. Pre-operative genetic and clinical characteristics of the STN-DBS study cohort. Table S2. Associations between polygenic risk score with baseline clinical and neuropsychological testing measures. Table S3. Associations between polygenic risk score with 3- and 24-month post-surgical clinical and neuropsychological testing measures. Figure S1. Polygenic risk score by genotypic subgroups. Figure S2. Polygenic risk score excluding GBA and LRRK2 variants by genotypic subgroups. [file 12883_2023_3188_MOESM1_ESM.docx]

## **Supplementary Table 1. Pre-operative genetic and clinical characteristics of the STN-DBS study cohort**

| **Characteristics** | **National Institutes of Health Parkinson’s Disease Clinic (n=33)** | **Parkinson’s Progression Markers Initiative (n=13)** |
| --- | --- | --- |
| Age at onset $\pm$ SE, years | 46.8$\pm$1.7 | 49.0 $\pm$ 2.2 |
| Disease duration $\pm$ SE, years | 12.9$\pm$ 1.1 | 7.9 $\pm$ 0.6 |
| Age at surgery $\pm$ SE, years | 59.8 $\pm$ 1.4 | 56.9 $\pm$2.1 |
| Sex | 21 M, 12 F | 5 M, 8 F |
| Motor Disability (MDS-UPDRS* part III) $\pm$ SE, score | 28.3 $\pm$ 1.5 | 25.3 $\pm$ 3.8 |
| Levodopa-equivalent daily dose, (mg/d) $\pm$ SE | 1034.24 $\pm$ 79.2 | NA |
| Cases with a pathogenic mutation, n/N (% of whole group) | 4/33 (12) | 6/13 (46) |
| PD-related genetic mutations (number of cases) | *GBA* N370S (1), *GBA* R159W (2), *GBA* T369M (2), *PRKN* E5-E7 deletion and frameshift mutation (1) | *GBA* N370S (1), *GBA* T369M (1), *LRRK2* G2019S (4) |

*Movement Disorder Society – sponsored revision of the Unified Parkinson’s Disease Rating Scale

**Supplementary Table 2. Associations between polygenic risk score with baseline clinical and neuropsychological testing measures**

| **Measure** | **Full PRS (n=33)** |  | **PRS excluding *GBA* and *LRRK2* variants (n=33)** |  | **PRS removing pathogenic and high risk variant carriers (n=27)** |  |
| --- | --- | --- | --- | --- | --- | --- |
|  | **Beta (SE)** | ***P*** | **Beta (SE)** | ***P*** | **Beta (SE)** | ***P*** |
| UPDRS I | -0.64 (0.84) | 0.45 | -0.46 (0.50) | 0.37 | -0.68 (1.01) | 0.51 |
| UPDRS II | -4.55 (2.65) | 0.10 | -2.87 (1.56) | 0.08 | -4.00 (3.11) | 0.21 |
| MDS-UPDRS* III | -3.97 (1.51) | 0.01 | -2.70 (1.47) | 0.07 | -5.77 (2.91) | 0.06 |
| UPDRS IV | 0.04 (0.73) | 0.95 | 0.00 (0.44) | 0.99 | 0.09 (0.86) | 0.92 |
| LEDD (mg/day) | 171.92 (81.96) | 0.05 | 152.85 (82.97) | 0.08 | 230.41 (79.32) | 0.01 |
| Mattis Dementia Rating Scale | 0.59 (0.71) | 0.41 | 0.61 (0.71) | 0.71 | 1.09 (0.80) | 0.18 |
| Beck Depression Inventory | -0.95 (0.89) | 0.30 | -0.95 (0.89) | 0.29 | -0.56 (0.84) | 0.51 |
| Phonemic Fluency | 1.20 (1.86) | 0.52 | 1.14 (1.85) | 0.54 | 0.49 (1.87) | 0.79 |
| Semantic Fluency | 2.04 (2.00) | 0.32 | 1.43 (2.01) | 0.48 | 1.80 (2.16) | 0.41 |

*NIH + PPMI combined cohort analysis. Full PRS (n=46), PRS excluding *GBA* and *LRRK2* variants (n=46), PRS removing pathogenic and high risk variant carriers (n=34)

**Supplementary Table 3. Associations between polygenic risk score with 3- and 24-month post-surgical clinical and neuropsychological testing measures**

| **Measure** | **Full PRS** | | | **PRS excluding *GBA* and *LRRK2* variants** | | | **PRS removing pathogenic and high-risk variant carriers** | | |
| --- | --- | --- | --- | --- | --- | --- | --- | --- | --- |
|  | **Beta (SE)** | ***P*** | **n** | **Beta (SE)** | ***P*** | **n** | **Beta (SE)** | ***P*** | **n** |
| UPDRS I: 3-month change | -0.62 (0.34) | 0.08 | 31 | -0.37 (0.20) | 0.09 | 31 | -0.959(0.44) | 0.38 | 25 |
| UPDRS I: 24-month change | -1.47 (0.71) | 0.05 | 27 | -0.93 (0.45) | 0.05 | 27 | -0.83 (0.71) | 0.26 | 23 |
| UPDRS II: 3-month change | 2.70 (2.02) | 0.19 | 30 | 1.65 (1.21) | 0.19 | 30 | 0.41 (2.33) | 0.86 | 25 |
| UPDRS II: 24-month change | 2.38 (4.05) | 0.56 | 26 | 1.50 (2.56) | 0.56 | 26 | 2.93 (4.94) | 0.56 | 23 |
| MDS-UPDRS III: 3-month change | -0.81 (1.67) | 0.63 | 45 | 1.09 (1.51) | 0.48 | 45 | 2.94 (3.05) | 0.34 | 34 |
| MDS-UPDRS III: 24-month change | -1.02 (3.58) | 0.78 | 27 | -0.65 (2.26) | 0.78 | 27 | 0.97 (2.51) | 0.70 | 23 |
| UPDRS IV: 3-month change | 1.32 (0.83) | 0.12 | 32 | 0.86 (0.50) | 0.10 | 32 | 1.20 (0.98) | 0.23 | 26 |
| UPDRS IV: 24-month change | -1.53 (0.86) | 0.09 | 27 | -0.97 (0.54) | 0.09 | 27 | -1.70 (0.99) | 0.10 | 23 |
| LEDD (mg/day): 3-month change | -19.20 (55.43) | 0.73 | 33 | -15.13 (54.41) | 0.78 | 33 | -28.16 (59.94) | 0.64 | 32 |
| LEDD (mg/day): 24-month change | 66.70 (64.03) | 0.31 | 30 | 58.98 (62.63) | 0.36 | 30 | 107.42 (68.71) | 0.13 | 30 |
| Mattis Dementia Rating Scale: 3-month change | 0.75 (0.94) | 0.43 | 32 | 0.87 (0.93) | 0.36 | 32 | 0.56 (1.06) | 0.60 | 31 |
| Mattis Dementia Rating Scale: 24-month change | 0.84 (1.26) | 0.51 | 27 | 0.52 (1.26) | 0.68 | 27 | 0.47 (1.64) | 0.77 | 27 |
| Beck Depression Inventory: 3-month change | -1.17 (0.81) | 0.16 | 30 | -1.36 (0.79) | 0.10 | 30 | -2.16 (0.85) | 0.02 | 30 |
| Beck Depression Inventory: 24-month change | -1.57 (1.24) | 0.22 | 27 | -2.34 (1.16) | 0.06 | 27 | -1.90 (1.01) | 0.07 | 27 |
| Phonemic Fluency: 3-month change | 0.46 (1.75) | 1.48 | 30 | -0.15 (1.47) | 0.92 | 30 | -0.99 (1.44) | 0.50 | 29 |
| Phonemic Fluency: 24-month change | -1.31 (2.01) | 0.52 | 27 | -1.38 (1.99) | 0.50 | 27 | -2.88 (2.22) | 0.21 | 26 |
| Semantic Fluency: 3-month change | 1.43 (2.02) | 0.48 | 31 | 1.49 (1.98) | 0.46 | 31 | -0.03 (2.28) | 0.99 | 30 |
| Semantic Fluency: 24-month change | 0.83 (2.56) | 0.75 | 27 | 0.76 (2.50) | 0.76 | 27 | -0.97 (2.67) | 0.72 | 25 |

Note: Follow-up data was not available for all participants, therefore, the available n is reported for each measure.

**Supplementary Figure 1. Polygenic risk score by genotypic subgroups**

**
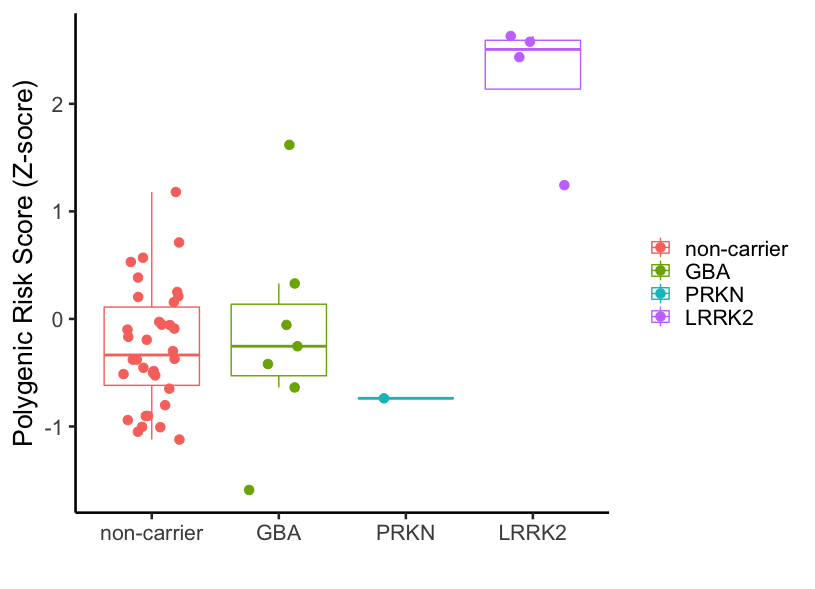
**

**Supplementary Figure 2. Polygenic risk score excluding *GBA* and *LRRK2* variants by genotypic subgroups**

**
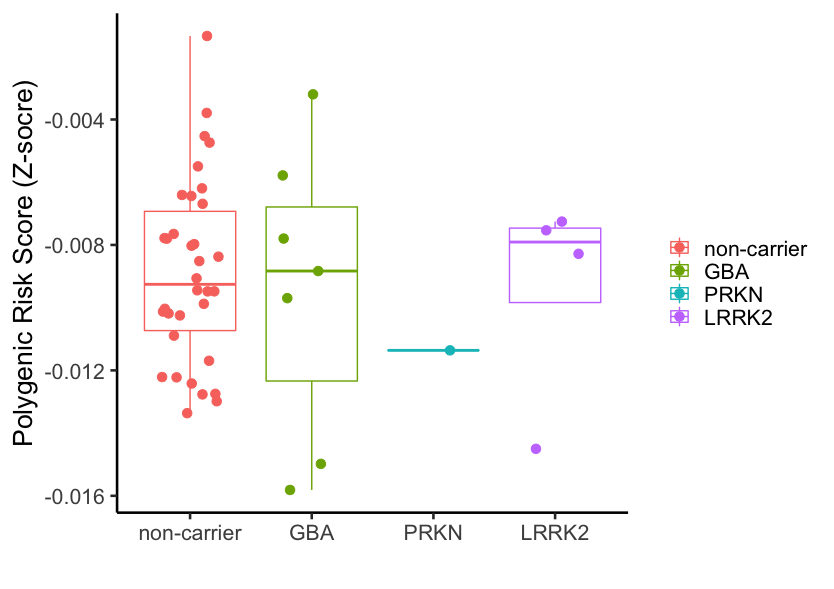
**
